# Oxidative Stress and the Central Nervous System

**DOI:** 10.3390/antiox14010110

**Published:** 2025-01-19

**Authors:** Marcello D’Ascenzo, Claudia Colussi

**Affiliations:** 1 Department of Neuroscience, Università Cattolica del Sacro Cuore, 00168 Rome, Italy; 2 Fondazione Policlinico Universitario Agostino Gemelli, IRCCS, 00168 Rome, Italy; 3 Istituto di Analisi dei Sistemi ed Informatica “Antonio Ruberti”, National Research Council, 00185 Rome, Italy

## 1. Introduction

Reactive oxygen and nitrogen species (ROS; RNS) are natural bioproducts of cellular metabolism, particularly produced within the mitochondria during energy production. These molecules play important roles in local cell signaling and homeostasis at low levels and are tightly controlled by antioxidant defenses [1].

The antioxidant function in cells, performed both enzymatically and non-enzymatically, is essential to the body’s defense system by maintaining the redox balance. Key enzymatic antioxidants include superoxide dismutase (SOD), catalase, and glutathione peroxidase, which work synergistically to scavenge and neutralize ROS. Non-enzymatic antioxidants such as glutathione, vitamins C and E, and flavonoids also play a vital role by directly interacting with free radicals. Nevertheless, imbalance between ROS/RSN production and the body’s ability to neutralize them with antioxidants occurs under many pathophysiological conditions leading to their accumulation and oxidative damage to lipids, proteins, and DNA [2]. Efficient protein turnover, which helps to keep the amount of damaged deleterious proteins under control, is especially crucial for post-mitotic cells such as neurons. However, neurons and many of the proteins inside these cells are long-lived structures and, as such, are particularly prone to oxidative stress accumulation [3].

The nervous system is particularly vulnerable to oxidative stress owing to its high metabolic activity, abundant lipid content, and relatively low levels of endogenous antioxidants [4]. In neurodegenerative diseases like Alzheimer’s disease [5,6], Parkinson’s disease, amyotrophic lateral sclerosis, and Huntington’s disease [7], oxidative stress contributes to neuronal damage. Indeed, in these devastating diseases, oxidative stress leads to several cellular dysfunctions, including protein misfolding, activation of glial cells, impairment of mitochondrial function, and, ultimately, programmed cell death. Similarly, oxidative stress exacerbates outcomes in acute conditions such as stroke, traumatic brain injury, and multiple sclerosis, where inflammation and ischemia drive ROS production. In addition, oxidative-stress-dependent mechanisms are crucial in mediating the detrimental effects of environmental factors (pollution, smoke, metals, etc.), chemotherapeutic treatments, or cerebrovascular alterations leading to life-threatening neurological conditions.

This Special Issue, “Oxidative Stress and the Central Nervous System”, includes five original research articles and three review papers, each addressing the contribution of oxidative-stress-mediated mechanisms to neuronal dysfunction in the context of degenerative diseases or secondary to environmental/internal stressors. These findings could potentially impact novel therapeutic approaches for the treatment of oxidative-stress-related CNS diseases.

## 2. Overview of Published Articles

### 2.1. Evaluating the Neuroprotective Potential of Caffeinated Coffee in the Context of Aluminum-Induced Neurotoxicity: Insights from a PC12 Cell Culture Model

Many environmental factors, including metals, may induce neurotoxicity through the production of oxidative stress. In this context, Rodak and colleagues discuss the role of aluminum, one of the most abundant metallic elements that is widely used as a component of readily available products including food, drinking water, and cosmetics. While the absorption of Al in its insoluble form is low, Al derivatives are instead absorbed and can cross the blood–brain barrier, and it is thought that they may induce neurodegeneration either by provoking protein misfolding, aggregation, and oligomerization or inducing oxidative stress. In an in vitro model, the authors show that caffeinated products can reduce Al-dependent toxicity, at least in part, by reducing oxidative stress. Discussion is also provided regarding an additional protective mechanism of caffeine products based on the chelation of aluminum that prevents Al from entering the cell.

### 2.2. Impact of Maternal Environment and Inflammation on Fetal Neurodevelopment

The contribution of Lubrano and colleagues provides an overview of the importance of inflammation and oxidative stress as common effectors of environmental stressors to early fetal and neonatal brain development. Indeed, many environmental insults induce a maternal inflammatory state that, possibly through epigenetic mechanisms, alters the fetal nutrient supply and energetic balance through the production of oxidative stress, with negative consequences for brain organization and development. Emphasis is given to many factors alone or in combination able to elicit or enhance maternal inflammation such as dietary patterns before or during pregnancy, air pollution, smoke, stress, depression, and anxiety. The early identification of these environmental risk factors by healthcare professionals and clinicians could help to reduce adverse effects on brain growth and development during pregnancy.

### 2.3. Potential of Natural Phenolic Compounds Against Doxorubicin-Induced Chemobrain: Biological and Molecular Mechanisms Involved

A large number of cancer patients treated with anthracyclines develop not only significant short-term alterations in organ functions but also important long-term complications in brain function, a condition referred to as “chemobrain”. These survivor patients deal with important defects in daily activities and memory performance. Of note, anthracyclines, such as doxorubicin, are known to produce oxidative stress and inflammation. The work from Serini and Calviello critically analyzes the results of preclinical studies on the efficacy of natural phenolic compounds (PheCs) that have shown potent antioxidant and anti-inflammatory actions able to counteract the negative impact of doxorubicin on brain damage and cognition. Specifically, the authors thoroughly discuss doxorubicin’s mechanism and the action of PheCs that are able to reduce oxidative stress and inflammation and restore physiological levels of neurotrophic factors and neurotransmitters.

### 2.4. Expression of ChAT, Iba-1, and nNOS in the Central Nervous System Following Facial Nerve Injury

In a rat model of peripheral facial nerve injury, Lee and colleagues show that facial nerve injury through compression or axotomy induces significant central nervous system impairment. They found that facial motor alterations are mainly due to a decrease in the expression of ChAT, the main enzyme that synthesizes the important neurotransmitter acetylcholine that is released at the neuromuscular junction in order to activate muscles. Furthermore, these damages were associated with an increased level of IBA-1, indicating an inflammatory response, and with the expression of nNOS. While the production of nitric oxide from neuronal nitric oxide synthase (nNOS) may contribute to neuroprotection and repair mechanisms, the authors conclude that a prolonged expression may instead be deleterious, sustaining oxidative stress and inflammation.

### 2.5. Administration of Bicarbonate Protects Mitochondria, Rescues Retinal Ganglion Cells, and Ameliorates Visual Dysfunction Caused by Oxidative Stress

With their contribution, Bastola et al. established an important link between the sAC/cAMP/PKA axis and mitochondrial regulation in an experimental model of glaucoma. Upstream activation of the soluble adenylyl cyclase by bicarbonate modulates the cAMP/PKA signaling pathway, preserving mitochondrial dynamics, bioenergetics, and biogenesis. In an experimental model of oxidative stress, bicarbonate administration was able to protect retinal ganglion cells and to improve visual function while decreasing glial activation. sAC-mediated mitochondrial protection could be a therapeutic approach for treating glaucoma.

### 2.6. NHE1 Protein in Repetitive Mild TBI-Mediated Neuroinflammation and Neurological Function Impairment

A fraction of patients with mild traumatic brain injury (mTBI) develop persistent post-concussion symptoms associated with long-term cognitive deficits and diffuse axonal damage. It is thought that astrocyte reactivity, microglial activation, and increased oxidative stress lead to morphological and gene expression changes that characterize this pathology. New findings from Bielanin and colleagues support the crucial role of the Na+/H+ exchanger protein (NHE1) in the inflammatory state, morphological alteration, impaired motor learning, and spatial memory observed in mice subjected to mTBI. Interestingly, the administration of the NHE1 inhibitor HOE642 reversed motor and cognitive deficits, along with gliosis, oxidative stress, and axonal damage.

### 2.7. Integrative Human Genetic and Cellular Analysis of the Pathophysiological Roles of AnxA2 in Alzheimer’s Disease

Much research has focused on the identification of valuable biomarkers for the early detection of AD, optimization of emerging treatments, or prediction of disease progression. Through bioinformatic analysis, using a microarray dataset available for human AD and healthy controls, the work from Ye and colleagues identifies the gene AnxA2 as a potential hub for AD pathology and mitochondrial function. Indeed, transcriptomic analysis revealed that oxidative phosphorylation, the cell cycle, AD-related genes, protein processing, vesicle transport, and autophagy were all affected in cells with the knockdown of AnxA2. Furthermore, reduced levels of AnxA2 directly influenced the accumulation of Aβ42 and the downstream negative effects on cell function.

### 2.8. Interactions Between Ferroptosis and Oxidative Stress in Ischemic Stroke

In their review, Liu and colleagues give an overview of the role of ferroptosis, a newly discovered programmed cell death dependent on iron and the accumulation of lipid peroxides, in ischemic stroke. The authors discuss the role of oxidative stress as a main determinant of ferroptosis and their mutual interplay in the establishment of a vicious inflammatory circle that contributes to ischemic stroke. Given that inhibition of ferroptosis drastically reduces brain tissue damage following cerebral ischemia, attention is also focused on the development of inhibitors of ferroptosis as a potential therapeutic approach.
